# *Quillaja brasiliensis* saponin-based nanoparticulate adjuvants are capable of triggering early immune responses

**DOI:** 10.1038/s41598-018-31995-1

**Published:** 2018-09-11

**Authors:** Samuel Paulo Cibulski, Mariana Rivera-Patron, Gustavo Mourglia-Ettlin, Cecilia Casaravilla, Anna Carolina Alves Yendo, Arthur Germano Fett-Neto, José Alejandro Chabalgoity, María Moreno, Paulo Michel Roehe, Fernando Silveira

**Affiliations:** 1 Departamento de Microbiologia, Laboratório de Virologia, Universidade Federal do Rio Grande do Sul (UFRGS), Porto Alegre, RS Brazil; 2 Departamento de Desarrollo Biotecnológico. Instituto de Higiene – Facultad de Medicina, Universidad de la República (UdelaR). Av. Alfredo Navarro 3051. CP., 11600 Montevideo, Uruguay; 3 Área Inmunología, Departamento de Biociencias/Instituto de Química Biológica – Facultad de Química/Ciencias, Universidad de la República (UdelaR). Av. Alfredo Navarro 3051. CP., 11600 Montevideo, Uruguay; 4 Laboratório de Fisiologia Vegetal, Centro de Biotecnologia e Departamento de Botânica, Universidade Federal do Rio Grande do Sul (UFRGS), Porto Alegre, Rio Grande Do Sul Brazil; 5 Present Address: Laboratório de Biología Celular e Molecular. Centro de Biotecnologia - CBiotec., Universidade Federal da Paraíba. Cidade Universitária, CEP 58051-900 João Pessoa, Paraíba Brazil

## Abstract

Commercially available saponins are extracted from *Quillaja saponaria* barks, being Quil A^®^ the most widely used. Nanoparticulate immunostimulating complexes (ISCOMs or ISCOMATRIX) formulated with these, are able to stimulate strong humoral and cellular immune responses. Recently, we formulated novel ISCOMs replacing QuilA^®^ by QB-90 (IQB-90), a *Quillaja brasiliensis* leaf-extracted saponin fraction, and reported that IQB-90 improved antigen uptake, and induced systemic and mucosal antibody production, and T-cell responses. However, its mechanism of action remains unclear. In this study we provide a deeper insight into the immune stimulatory properties of QB-90 and ISCOMATRIX-like based on this fraction (IMXQB-90). We show herein that, when used as a viral vaccine adjuvant, QB-90 promotes an “immunocompetent environment”. In addition, QB-90 and IMXQB-90 induce immune-cells recruitment at draining-lymph nodes and spleen. Subsequently, we prove that QB-90 or IMXQB-90 stimulated dendritic cells secret IL-1β by mechanisms involving Caspase-1/11 and MyD88 pathways, implying canonical inflammasome activation. Finally, both formulations induce a change in the expression of cytokines and chemokines coding genes, many of which are up-regulated. Findings reported here provide important insights into the molecular and cellular mechanisms underlying the adjuvant activity of *Q*. *brasiliensis* leaf-saponins and its respective nanoparticles.

## Introduction

Vaccination has been one of the most effective tools to reduce morbimortality caused by infectious diseases. A number of vaccines often require the addition of adjuvants to induce adequate immunological stimuli and to achieve protection upon challenge. Adjuvants have been used in veterinary and human vaccines for almost a century in attempting to increase vaccine immunogenicity, largely by activating innate immunity, promoting controlled inflammation and enhancing adaptive immune responses. Limiting residual toxicity and adverse side effects of induced inflammation is a major hurdle for adjuvant use in human vaccines^1^, whose mitigation is usually hampered by a limited understanding of their mechanism of action^2–4^. That is why very few adjuvants are licensed for human use; yet, several formulations are being evaluated in clinical trials^5,6^.

Evidence has been accumulated suggesting that adjuvants must activate the innate immune system, as a prerequisite for generating a robust and protective adaptive response^7^. Therefore there is still a need for designing novel adjuvants able to adequately modulate the global immune response^3,8,9^. Triterpenoid saponins, such as Quil A^®^, extracted from *Quillaja saponaria* Molina, have been widely used as adjuvants for many years in several vaccines of veterinary use^10^. Although these compounds are able to trigger strong cellular and humoral immune responses, their use in human vaccines has been restricted due to undesirable side effects, such as local reactions, haemolytic activity and systemic toxicity^10,11^. A more purified saponin fraction, also extracted from the bark of the *Q*. *saponaria*, QS-21 is currently considered “the gold standard” molecule in terms of saponin-based adjuvants^12^, is being assessed in clinical trials due to its ability in tailoring Th1-biased immune responses, leading to protection against cancers and intracellular pathogens^13–16^. However, the mechanism of action by which QS-21 activates cells remains unknown, and the signaling pathways it induces are also still poorly understood^2–4,12^.

Throughout the last decade our group has been studying the adjuvant properties of saponins from the leaves of *Quillaja brasiliensis* (A. St.-Hil. etTul.) Mart., a native tree from southern Brazil and Uruguay. Several leaf saponin fractions from *Q*. *brasiliensis* were found to share structural and biological activities with *Q*. *saponaria*^17–20^, inducing lower levels of toxicity than Quil A^®^ in mice, and strongly potentiating humoral and cellular immune responses to viral antigens^18,20–23^. Indeed, these molecules were shown to up-regulate both Th1- and Th2-like immune responses and to promote a robust DTH reaction. QB-90 and QB-80, two of the *Q*. *brasiliensis* saponin fractions, were shown to increase secretion of Th1-associated cytokines (IFN-γ and IL-2) and antigen-specific IFN-γ production by CD4^+^ and CD8^+^ T cells^20,24^ when used as vaccine adjuvants.

In order to reduce the undesirable haemolytic activity that invariably accompanies the adjuvant effect of saponins, different colloidal preparations which can also act as antigen delivery systems have been formulated^9,25–27^. One of such preparations, called immunostimulating complexes (ISCOMs)^28^ are 40 nm cage-liked self-assembled structures combining Quil A^®^, cholesterol, phospholipids and antigen. A similar preparation, named ISCOMATRIX^TM^, is a vaccine adjuvant formulation which does not include the antigen^29–31^. The physical properties of ISCOM adjuvants contribute to antigen stability, reduce the haemolytic effects associated with saponins, interact with dendritic cells (DCs) and enhance cross-presentation of the incorporated antigen, generating both antibody and CD4^+^ and CD8^+^ T cell responses^2,29,31,32^. In summary, ISCOM and ISCOMATRIX^TM^ vaccines are known to induce long-lasting antibody responses, a balanced Th1/Th2 response, and generation of cytotoxic T lymphocytes in mice^33,34^ and humans^13,15,35^.

Recently, we reported an alternative ISCOM formulation replacing Quil A^®^ by QB-90 (IQB-90). This formulation with reduced haemolytic activity was efficiently uptaken *in vitro* by murine bone marrow-derived dendritic cells (BMDCs). Moreover, subcutaneously inoculated IQB-90 induced strong serum antibody responses to ovoalbumin, robust DTH reactions, significant T cell proliferation and increased Th1 (IFN-γ and IL-2) cytokine responses. Similarly, intranasally delivered IQB-90 elicited serum IgG and IgG1, and mucosal IgA responses at distal systemic sites, even with low antigen doses^34^. Despite the fact that ISCOMs and ISCOMATRIX^TM^ have been studied for nearly 30 years, the mechanisms of action of such nanoparticles are still not clearly understood^2,31,36^.

The understanding of saponin-based adjuvants mode of action, and in particular of those derived from *Q*. *brasiliensis* leaves, is highly relevant, as they constitute a more readily renewable alternative source of saponins and also present reduced toxicity compared to *Q*. *saponaria* bark- derived saponins. Therefore, the aim of the present work is to provide a deeper insight into the molecular mechanisms of action of QB-90 and its nanoparticle formulations. We will focus on the interaction with innate immunity receptors, as well as in the characterization of early immune events, such as cell recruitment and cytokine/chemokine profile, triggered by *Q*. *brasiliensis* saponin-based adjuvant.

## Results

### QB-90 saponins promote a transient immunocompetent environment lasting 24 hours regardless the adjuvant-antigen mixture

**Figure 1:**
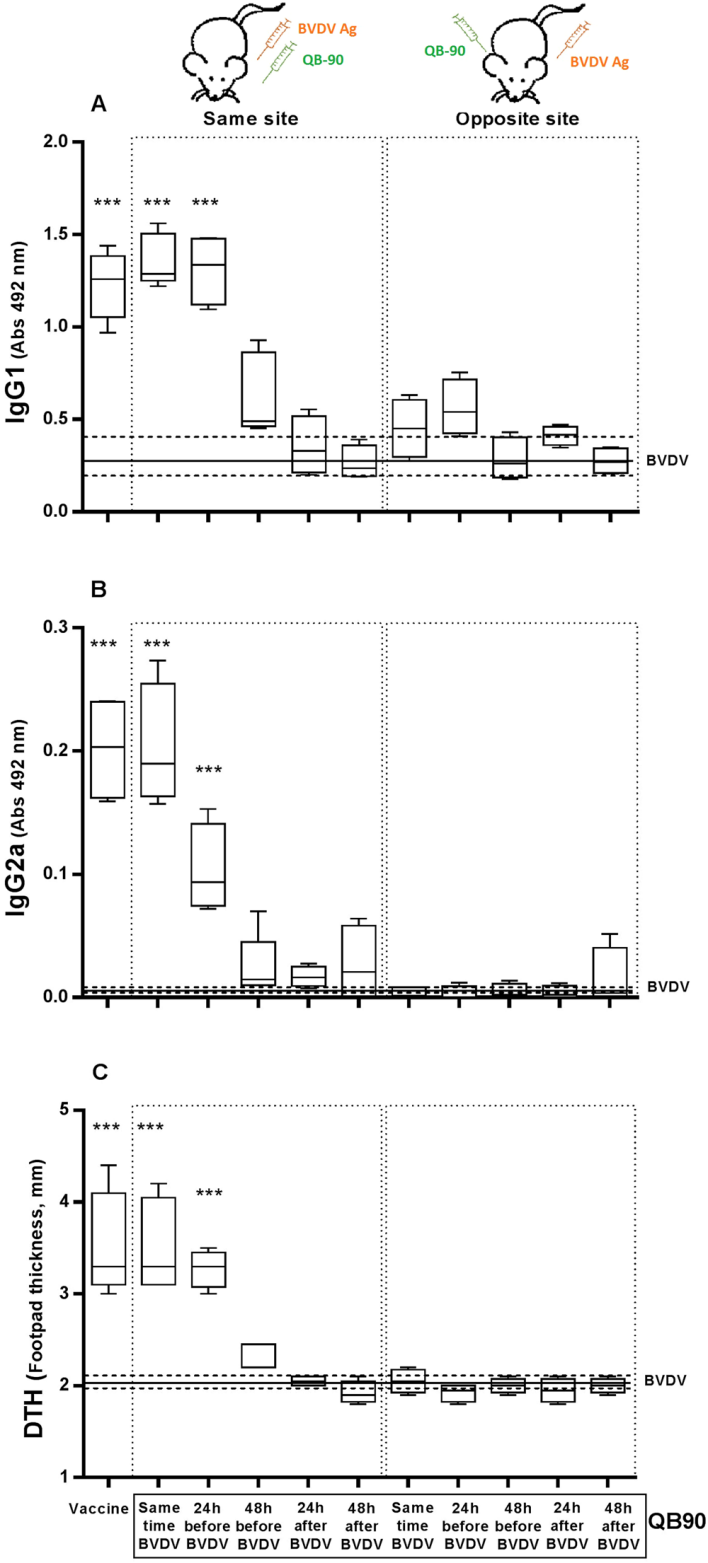
QB-90 adjuvant activity is independent of antigen-adjuvant mixture, but these should be administered in the same site. Female CF1 mice (*n* = 5) were subcutaneously immunized in the hind limb twice (0 and 14 day) at two weeks intervals with BVDV antigen and QB-90. Groups were inoculated with QB-90 and with BVDV antigen separately, either in the same or opposite sites, and at the same time or with different time points (BVDV antigen was inoculated simultaneously or 24 or 48 hours after the QB-90 inoculation). In addition, a group of mice was inoculated with the formulated vaccine (containing BVDV antigen and QB-90 as adjuvant). IgG1 (**A**) and IgG2a (**B**) anti-BVDV antibodies titers and DTH responses (**C**) at day 28 are shown in box-and-whiskers. Horizontal continuous lines represent median values in mice inoculated with BVDV alone, and dashed lines represent maximum and minimum values in the same group. Statistical analyses were performed using Kruskal-Wallis test (*** denotes *p* < 0.001, in relation to animals immunized with BVDV alone).

In order to assess if the mechanism of action of QB-90 was independent of the presence of antigen, BVDV antigen was injected in mice into proximal as well as distal sites, either at the same time or 24 and 48 hours before or after the administration of QB-90. As shown in Fig. 1, QB-90 was able to induce a strong immune response characterized by high antigen-specific antibody titers (IgG1 and IgG2a) and robust delayed type hypersensitivity (DTH) reaction, when administered at the same site 24 hours earlier or at the same time of the BVDV antigen. However, if the adjuvant was administered 48 hours before, or at distal injections sites, no adjuvant effect was observed. It is important to highlight, that no adjuvant effect was observed when QB-90 was administered 24 or 48 hours after the BVDV antigen administration (data not shown).

These findings suggest that the adjuvant QB-90 is able to trigger a transient (lasting 24 hours) and local immunocompetent environment that could be exploited by a subsequent administration of antigen. These data also suggest that QB-90 mechanism of action is independent of binding to BVDV antigen, but in order to trigger a systemic immune response, antigen should be administered up to 24 hours after the inoculation of QB-90, and in a proximal injection site.

### Increased cell numbers in spleen and dLNs after QB-90 or IMXQB-90 administration without antigen

**Figure 2:**
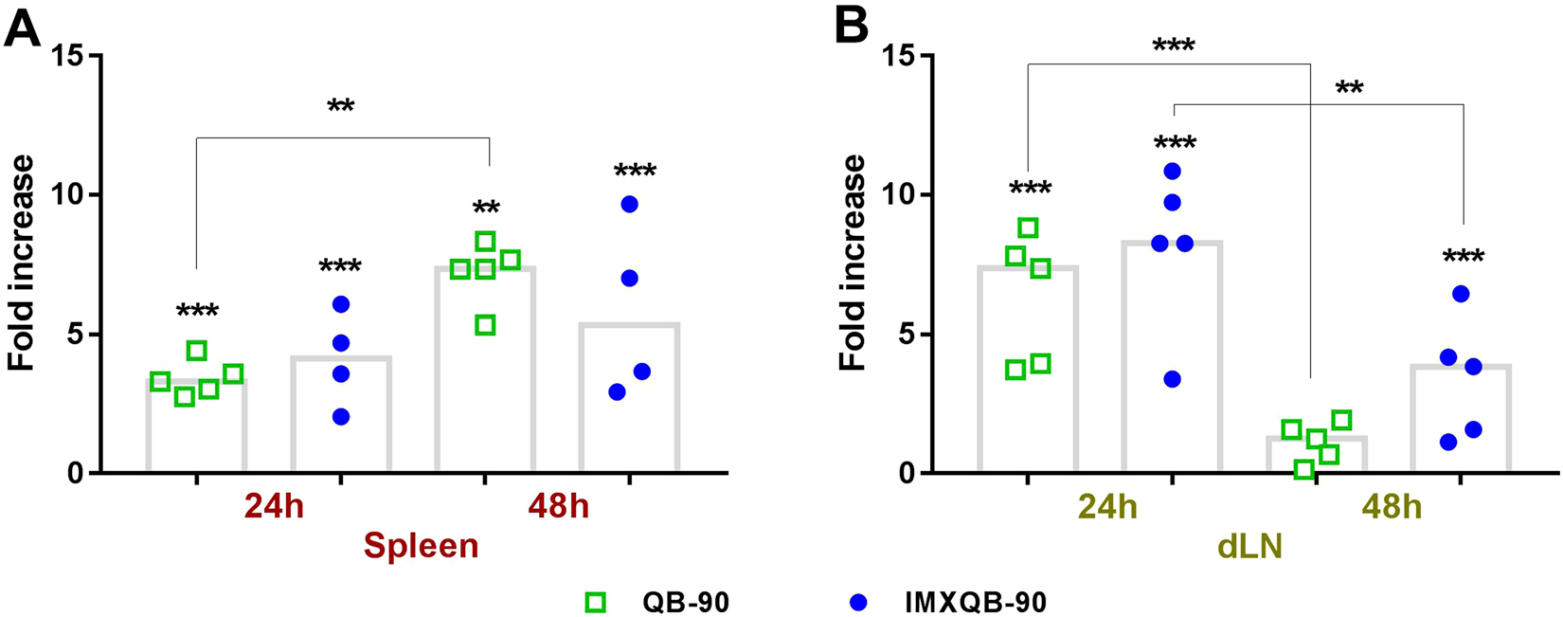
Cell count in spleen and dLNs of QB-90 and IMXQB-90 treated mice. Mice were injected at the base of the tail with QB-90 (10 µg), IMXQB-90 (2.5 µg of QB-90) or saline solution. After 24 and 48 hours post injection (hpi), cell counts were performed in spleen (**A**) and dLN (**B**), and expressed as fold increase regarding the saline inoculated group, using median values (*n* = 4–5) of each group. Statistical analysis was performed using Kruskal-Wallis test with Dunn’s post test. Statistically significant differences in cell numbers between QB-90, IMXQB-90 and saline-inoculated mice are indicated with (*p < 0.05), (**p < 0.01); (***p < 0.001).

At 24 and 48 hours post subcutaneous inoculation (hpi) of either QB-90 or IMXQB-90, single-cell suspensions were prepared from spleen and dLNs. Spleens from QB-90 and IMXQB-90-treated mice (Fig. 2A) showed a significantly increased number of cells when compared to mock controls at 24 and 48 hours (*p* < 0.001). However, when comparing 24 and 48 hpi among each group (QB-90 and IMXQB-90), statistically significant differences were observed only in the QB-90-inoculated group.

In dLNs from both QB-90 and IMXQB-90 treated mice (Fig. 2B), a significant increase in cell numbers was detected at 24 hpi (*p* < 0.001). However, at 48 hpi only the IMXQB-90-treated mice showed a significantly increased number of cells when compared to the control group (*p* < 0.01), although the number was significantly lower than that observed at 24 hpi.

### QB-90 and IMXQB-90recruits and activates immune cells in spleen and dLNs

**Figure 3:**
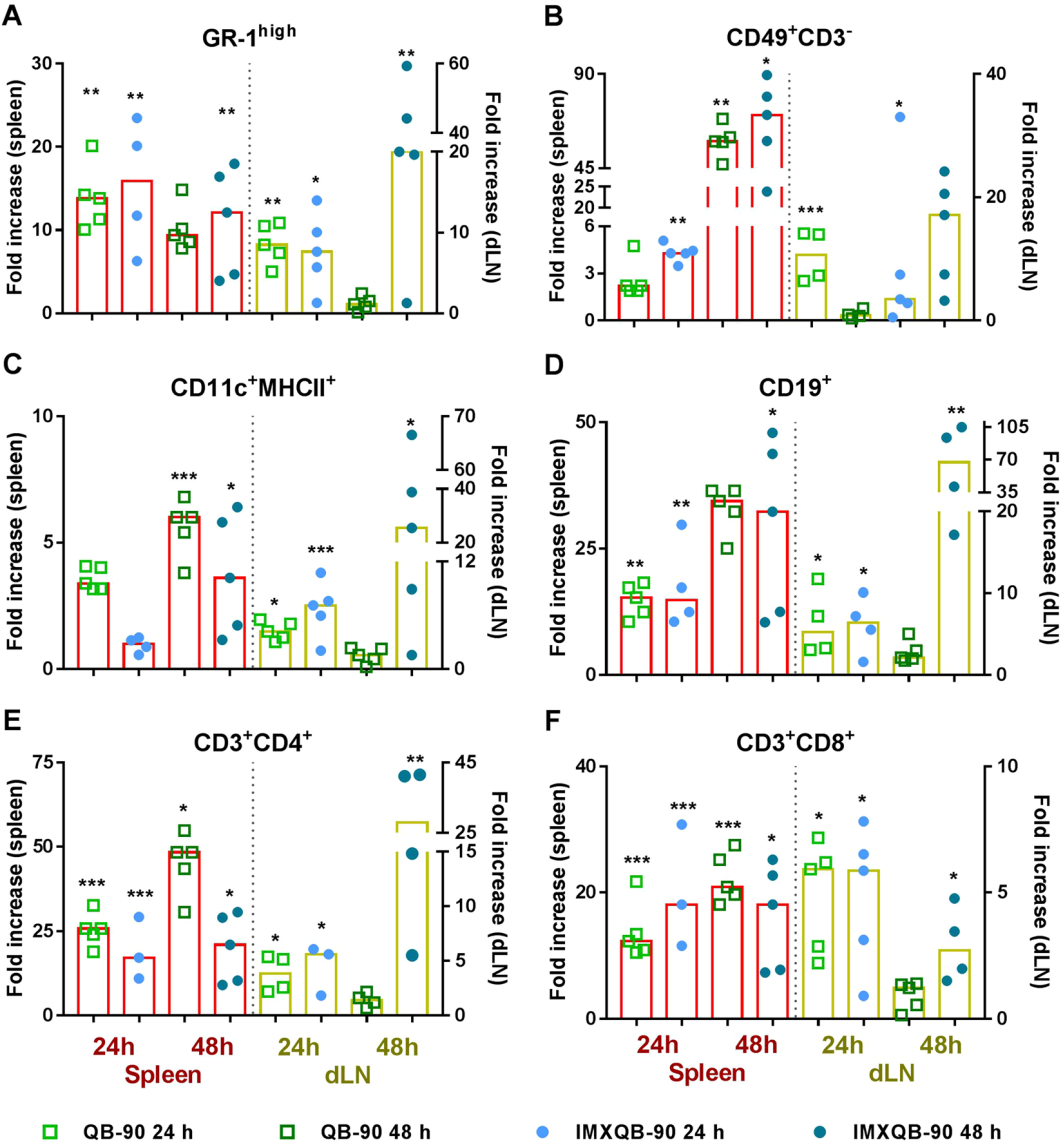
Cell populations in spleen and dLNs after inoculation of saponin-based adjuvants. QB-90 (10 µg), IMXQB-90 (2.5 µg of QB-90) or mock (saline solution) controls were subcutaneously injected at the base of the tail and at 24 and 48 hpi cells were collected and analyzed by flow cytometry. Spleen cell populations are shown in the left axis (red bars), and dLNs cell populations are shown in the left axis (yellow bars). Fold increase values regarding the mock control group were calculated using median values (*n* = 4–5). Statistically significant differences between adjuvants and saline treated mice were determined using Kruskal-Wallis test with Dunn’s post test, and are indicated with (*p < 0.05); (**p < 0.01); (***p < 0.001).

In spleen, neutrophils (Gr1^high^), Natural Killers (NK;CD49^+^CD3^−^), B cells (CD19^+^) and T cells (identified as CD3^+^CD4^+^ and CD3^+^CD8^+^) populations were higher in QB-90 and IMXQB-90-inoculated mice than in those of the mock (saline) inoculated group at 24 and 48 hpi (Fig. 3A–F left axis). Moreover, the dendritic cells (DCs) population, identified as CD11c expressing MHC class II, were significantly increased in IMXQB-90-treated mice, though only at 48 hpi (*p* < 0.05, Fig. 3C left axis).

In dLNs of mice that received either QB-90 or IMXQB-90, neutrophils, NKs, DCs, B cells and CD4^+^ and CD8^+^ T cells were significantly increased at 24 and 48 hpi (Fig. 3A–F right axis), with the exception of NK cells from the IMXQB-90 group at 48 hpi. Altogether, results showed that QB-90 and IMXQB-90-inoculations induce significant increase in cell numbers in both spleen and dLN.

Taken together, these results show that the early increased cell counts evidenced earlier is associated with an increase in neutrophils, NKs, DCs, B and T cell populations.

### Differential modulation of gene expression at dLN by QB-90 and IMXQB-90 vaccine adjuvants formulations

**Figure 4:**
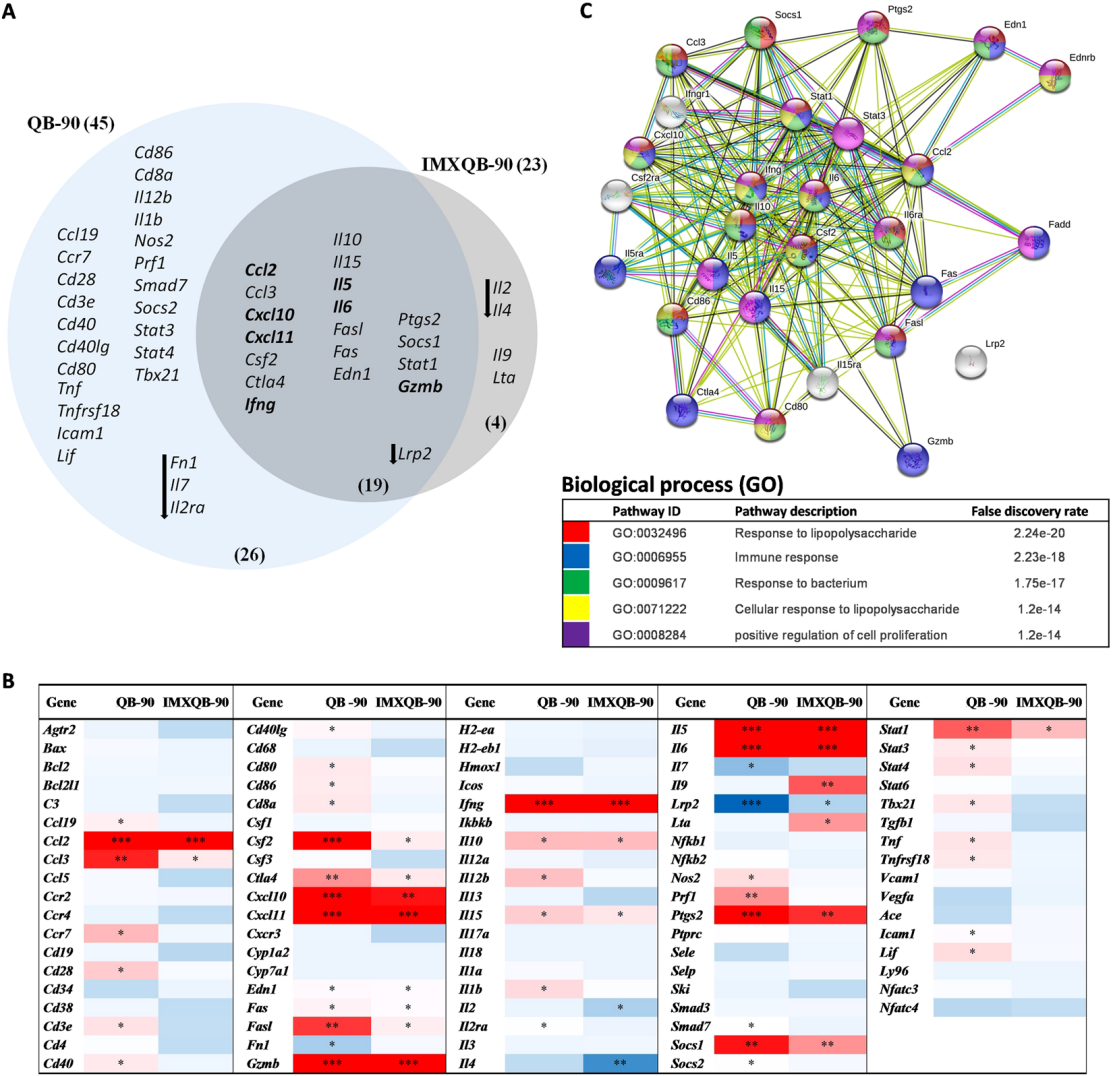
Cytokines and chemokines genes were up-regulated in the dLNs of mice 24 h after QB-90 and IMXQB-90 adjuvant administration. (**A**) Venn’s diagram showing the distribution of genes differentially expressed at least ≥two fold by QB-90 (*n* = 45) and IMXQB-90 (*n* = 23) treated groups were compared to a mock control (saline solution inoculated group). The total number of up-regulated genes (26, 19, 4) is indicated. The arrows show down-regulated genes. Genes up-regulated ≥10-fold are also bolded. (**B**) Heatmap of the differential gene expression analysis of the immune related genes in dLN of mice 24 h after QB-90 and IMXQB-90 adjuvant administration. RNA extracted from the dLN 24 h after injection of either QB-90- or IMXQB-90 were analyzed by qPCR (TaqMan^®^ Mouse Immune Array, Applied Biosytems). Red boxes: upregulation; blue boxes: downregulation. *refers to twofold variations (≥ or ≤) 2; **five-fold ≥5; ***ten-fold ≥10. (**C**) STRING network analysis of genes differentially expressed in dLNs of mice after QB-90 and IMXQB-90 administration. The identified genes from Table 1 were entered into the STRING database (https://string-db.org/). Proteins are represented as nodes. Node colors represent the different types of pathways in which proteins are involved (showed at the bottom of the figure). The STRING confidence level was set to 0.4 (medium).

The expression of ninety two genes related to immune function in dLN was analyzed by qPCR (TaqMan^®^ Mouse Immune Array Applied Biosystems) 24 hpi with QB-90, IMXQB-90 or saline solution as mock control. In the QB-90-treated group 45 genes were differentially expressed whereas in IMXQB-90-treated group these amounted to 23, in comparison with the mock group, as shown in the Venn’s diagram (Fig. 4A) and in the gene-expression heatmap (Fig. 4B).

The most prominent gene differential expression was detected in QB-90-treated mice, and involved the up-regulation of *Ccr7*, *Cd28*, *Il-12b*, *Il-1β*, *Nos2*, *Prf1* and *Lif* and the down-regulation of *Il-7*and *Fn1*. In addition, and up-regulation of several genes coding for the costimulatory molecules, such as *Cd40*, *Cd40lg*, *Cd80* and *Cd86*, was noticed.

In IMXQB-90 inoculated mice, only four genes showed differential expression in relation to QB-90 inoculated mice gene expression: *Il-*2 and *Il-*4 were down-regulated while *Il-9* and *Lta* were up-regulated. Nineteen genes were found differentially expressed in both groups (Table 1). Regarding the chemokine gene expression, three genes were found to be up-regulated in both treated groups: *Ccl2*, *Ccl3*, and *Cxcl10*. In addition, *Csf2*, *Ctla4*, *Ifn-γ*, *Il-10*, *Il-15*, *Il-5*, *Il-6* and *Fasl* expression was found to be increased (Table 1). Six other genes were up-regulated: *Fas*, *Edn1*, *Ptgs2*, *Socs1*, *Stat1* and *Gzmb*. On the contrary, *Lpr2* was down-regulated in both treated groups. In order to better underst and the molecular and cellular interactions between the differentially expressed genes in both groups we analyzed them as a query gene set using the web-based network tool STRING. Results indicate that nearly all genes differentially expressed in the context of QB-90 and IMXQB-90 inoculation are part of well-defined interaction networks (Fig. 4C). The analysis clearly shows that our proteins have more interactions among themselves than what would be expected for a random set of proteins (Protein-protein interactions enrichment p-value: <1.0e-16). The STRING analysis revealed the functional links between different proteins involving functions as “response to LPS” (GO:0032496), “immune response” (GO:0006955), “response to bacterium” (GO:0009617), “cellular response to LPS” (GO:0071222) and “positive regulation of cell proliferation” (GO:0008284). All cellular processes were identified with high confidence, as predictions showed a low false discovery rate (<1.47e-14).

**Table 1:** Genes differentially expressed in both treatments: QB-90 and IMXQB-90.

Gene	Gene name	Group	QB-90*	IMXQB-90*
*Ccl2*	chemokine (C-C motif) ligand 2	Chemokine	17,2	12,5
*Ccl3*	chemokine (C-C motif) ligand 3		8,7	2,6
*Cxcl10*	chemokine (C-X-C motif) ligand 10		15,2	9,2
*Cxcl11*	chemokine (C-X-C motif) ligand 11	Cytokine	69,8	32,4
*Csf2*	colony stimulating factor 2 (granulocyte-macrophage)		10,3	2,5
*Ctla4*	cytotoxic T-lymphocyte-associated protein 4		5,1	2,6
*Ifng*	interferongamma		20,4	11,3
*Il10*	interleukin 10		3,9	3,9
*Il15*	interleukin 15		3,4	2,7
*Il5*	interleukin 5		47,2	100,7
*Il6*	interleukin 6		22,9	28,3
*Fasl*	Fas ligand (TNF superfamily, member 6)		7,9	2,5
*Fas*	Fas (TNF receptor superfamily member 6)	Cytokine receptor	2,3	2,2
*Edn1*	endothelin 1	Peptidehormone	2,2	2,2
*Ptgs2*	prostaglandin-endoperoxidesynthase 2	Synthase	16,6	8,2
*Socs1*	Suppressorofcytokinesignaling 1	Kinasemodulator	9,2	5,1
*Stat1*	signal transducer and activator of transcription 1	Transcriptionfactor	6,7	3,9
*Gzmb*	granzyme B	Serine protease	42,5	10,8
*Lrp2*	low density lipoprotein receptor-related protein 2	Unclassified	−13,1	−2,0

*Refers to fold change variations.

Considering these results as a whole, QB-90 and IMXQB-90 were able to induce a differential expression pattern of cytokines, chemokines and costimulatory molecules which are particularly relevant in triggering early immune responses in dLNs, and thus, for creating a proinflamatory environment which is essential to orchestrate an effective and long-lasting immune response.

### QB-90 and IMXQB-90 promote a Th1-type cytokine profile

**Figure 5:**
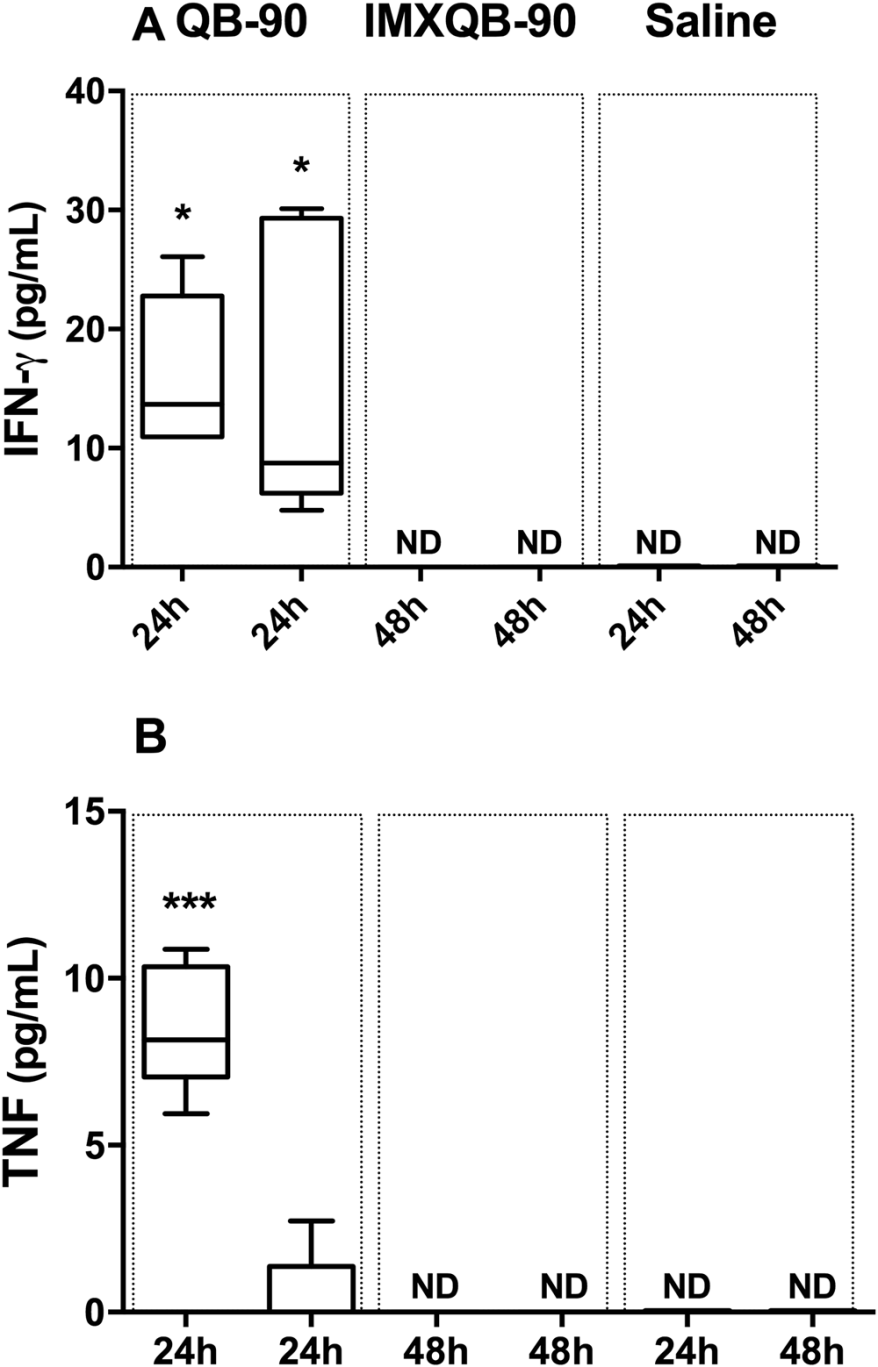
IFN-γ and TNF-α production in mice sera at 24 and 48hpi. IFN-γ (**A**) and TNF-α (**B**) production were measured by a Cytometric Bead Array (BD) 24 and 48 hours post-inoculation with QB-90 (10 µg) or IMXQB-90 (2.5 µg) in sera of sensitized mice. Cytokine levels expressed in pg/mL are shown in box-and-whiskers, and median of each group is indicated by a line. Statistically significant differences between adjuvants and saline treated mice were determined using Kruskal-Wallis test with Dunn’s post test and are indicated with (*p < 0.05); (***p < 0.001).

To better characterize the immunocompetent status elicited by QB-90 and IMXQB-90, the cytokine profile induced by these formulations was assessed in mice. Cytokines obtained from sera at 24 and 48 hpi were analyzed using a Th1/Th2/Th17 Cytokine Bead Array (BD). Although IL-2, IL-4, IL-6, IFN-γ, TNF-α, IL-17 and IL-10 production levels were assessed, only IFN-γ and TNF-α were detectable in sera obtained at 24 hpi. Similar levels of INF-γ were found in either QB-90 or IMXQB-90-sensitized mice (Fig. 5A, *p* < 0.01). TNF-α was significantly increased only in QB-90-inoculated mice (Fig. 5B, *p* < 0.05). None of the assessed cytokines were detected at 48 hpi.

### QB-90 and IMXQB-90 induce IL-1β production by BMDCs*in vitro* in a Caspase-1/11 dependent manner

**Figure 6:**
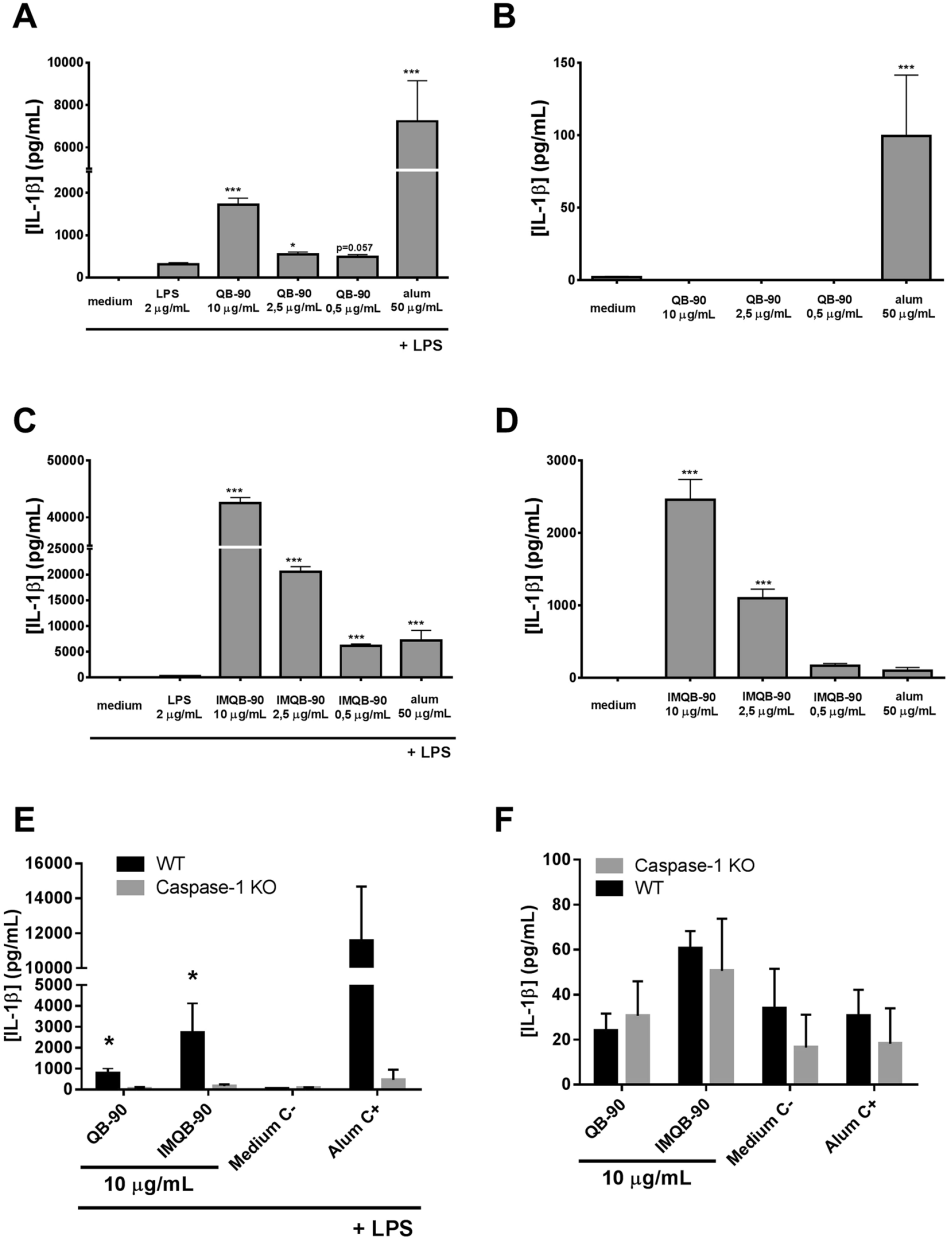
Induction of IL-1β secretion by QB-90 and IMQB-90 in murine BMDCs. WT C57Bl/6 mice BMDCs (**A**–**D**) were primed for two hours with (**A**,**C**) or without (**B**,**D**) 10 μg/mL of LPS, and then stimulated during three hours with 10, 2.5 y 0.5 µg/mL of either QB-90 (**A**,**B**) or IMXQB-90 (**C**,**D**). WT and caspase-1/11 KO C57Bl/6 mice BMDCs were primed with (**E**) or without (**F**) LPS (10 μg/mL), and then stimulated with 10 µg/mL of either QB-90 or IMXQB-90. In all cases, cells were stimulated with cell culture media or aluminum hydroxide (alum 50 μg/mL) as negative and positive controls, respectively. IL-1β was measured in culture supernatants by ELISA. Data are expressed as mean of triplicates ± SEM and are representative of two or more independent experiments. (*p < 0.05), and (***p < 0.001) indicate significant differences detected between negative control and adjuvant stimulated cells determined by student’s T- test.

BMDCs were obtained from C57BL6 WT mice and primed with the TLR4-agonist LPS (10 ng/mL) during 2 hours. Then, the cells were stimulated with increasing concentrations of QB-90 or IMXQB-90(10, 2.5 and 0.5 µg/mL) and the production ofIL-1β was analyzed in cell culture supernantants. In the presence of LPS, both QB-90 and IMXQB-90 (10 and 2.5 µg/mL) enhanced the production of IL-1β, suggesting that both preparations activate the canonical inflammasome pathways (Fig. 6A,C). It is worth to mention that after stimulation with alum (50 µg/mL), a known NLRP3 inflammasome activator used as control, IL-1β production levels were lower than after IMXQB-90 stimulation in lower doses (Fig. 6D).

Interestingly, in the absence of LPS, QB-90 was not able to induce the IL-1β production at none of the assessed concentrations (Fig. 6B) while IMXQB-90 induced a high production of the cytokine (both at 10 and 2.5 µg/mL; Fig. 6D). Cell viability after incubation remained unchanged (≥90%) in all cases (data not shown).

Then, we sought to determine if the IL-1β production induced by both QB-90 and IMXQB-90 was a consequence of the canonical inflammasome pathways activation. To that end, BMDCs derived caspase-1/11 KO and WT C57BL/6 mice were stimulated with 10 µg/mL of QB-90 or IMXQB-90 after 2 hours priming with LPS (10 ng/mL), and IL-1β production was measured. In the presence of LPS both QB-90 and IMXQB-90 (10 µg/mL) induced the production of IL-1β in WT BMDCs, nonetheless, this production was not seen in caspase-1/11 KO BMDCs (Fig. 6E). No differences were observed among groups when cells were not primed with LPS (Fig. 6F). These results indicate that the observed production of IL-1β is dependent on Caspase-1/11, and that both preparations would be able to activate canonical inflammasome pathways^37^.

### QB-90 and IMXQB-90induce proinflammatory responses through MyD88 pathways in a TLR-independent manner

In order to investigate the mechanisms by whichQB-90 and IMXQB-90 trigger proinflammatory responses, reporter cell lines Raw Blue™ expressing many pattern recognition receptors (PRR), THP1-XBlue™, expressing functional myeloid differentiation primary response 88 (MyD88) adaptor molecule, and THP1-XBlue™ defMyD cell-line (expressing only non-functional MyD88) were stimulated with the adjuvant preparations.

**Figure 7:**
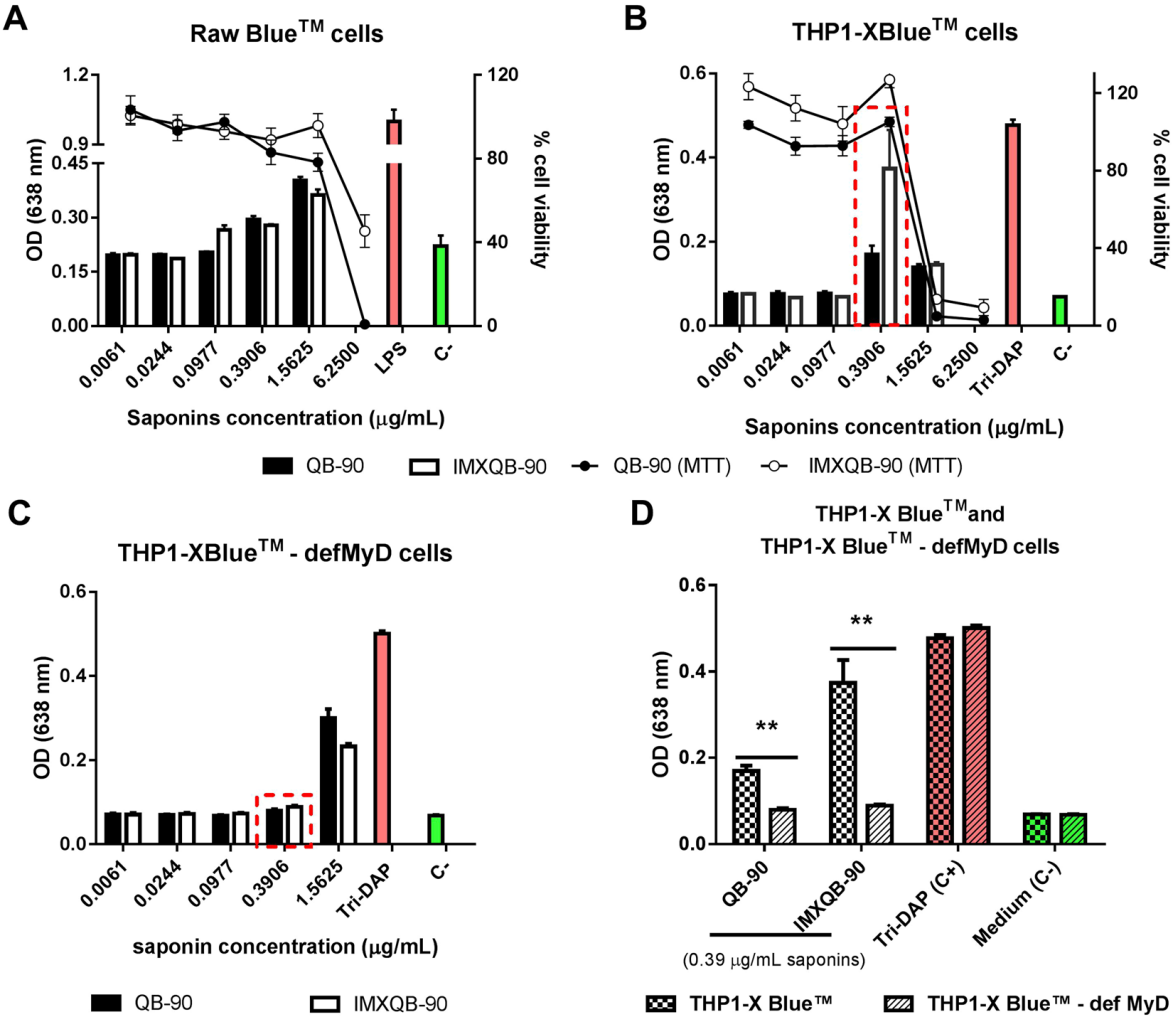
Reporter cell lines stimulated with QB-90 or IMXQB-90. Raw Blue^TM^ (**A**), THP-1XBlue^TM^ (**B**) and THP1-XBlue™-defMyD (**C**) reporter cell lines were stimulated overnight with either QB-90 or IMXQB-90 (containing 1.56, 0.39, 0.098, 0.024 or 0.006 μg of saponin per mL). Bars show expression of reporter gene (SEAP), quantified by optical density (OD) at 638 nm. The cell viability percentage, determined by MTT assay, is shown in lines and indicated in the right Y axis for Raw Blue^TM^ cells (**A**) and for THP-1 Blue^TM^ (**B**). In (**D**) a comparison of reporter-gene expression between THP-1 Blue^TM^ and THP1-XBlue™-defMyD at 0.39 μg/mL of saponins was performed. Cells were primed with LPS (1 µg/mL, Sigma) or Tri-DAP (10 μg/mL, InvivoGen^®^) as a positive control for Raw Blue^TM^ and both THP-1 cell lines, respectively. In B and C, data were analyzed by One-way ANOVA and Dunnet’s post test, comparing groups against the negative control (C-). In (**D**) data were analyzed by One-way ANOVA and t-test. Differences were considered statistically significant when P value < 0.05 (**P < 0.01).

As shown in Fig. 7A, none of the tested concentrations (100% of cell viability) for both adjuvant formulations showed interaction with any of the PRRs expressed by Raw Blue™ cells. In order to evaluate whether QB-90 or IMXQB-90 may signal through MyD88, THP-1 and THP1-XBlue™-defMyD reporter cell lines were stimulated with either QB-90 or IMXQB-90. Both formulations were able to activate the reporter gene (SEAP) in THP1-XBlue™ at 0.39 and 1.56 μg/mL (Fig. 7B). However, stimulation of THP1-XBlue™-defMyD occurred only at a concentration of 1.56 μg/mL (Fig. 7C). Of note, cell viability was assessed by MTT assay and 100% of viability was observed up to 0.39 μg/mL of saponins. Hence, the activation observed in both cell lines at 1.56 μg/mL can be attributed to cell cytotoxicity and the subsequent induction of danger signals (DAMPs) that augment immune responses^1,2^ (right Y axis Fig. 7A,B). As shown in Fig. 7D, when comparing the signal elicited in THP1-XBlue™ and THP1-XBlue™-defMyD cell lines stimulated with 0.39 μg/mL (100% of viability) of either QB-90 or IMXQB-90, it becomes evident that these formulations were able to activate the reporter gene in THP-1 cells, but not in cells lacking the MyD88 adaptor molecule. Altogether, these results suggest that the both *Q brasiliensis* saponin based formulations interact with monocytes or macrophages in a TLR-independent but MyD88-dependent manner.

## Discussion

Adjuvants are essential components of modern vaccines since they contribute to the initiation of the innate immune response to antigens^3,7^. Furthermore, they stimulate the production of cytokines by resident innate cells, which in turn leads to the rapid recruitment of neutrophils, monocytes and dendritic cells (DCs) that uptake and present antigens^35,38,39^. Adjuvant potential of plant-derived compounds has been largely explored. In particular, saponins (e.g., QS-21) or saponin-based formulations as ISCOMs or ISCOMATRIX^TM^ have shown to be safe in animal and human clinical studies and to be well tolerated by humans^13,27,35,40^. When administered subcutaneously, such adjuvants have shown to trigger powerful immune responses, characterized by the induction of CD4^+^ and CD8^+^ T cells, balanced Th1/Th2 cytokine responses, as well as robust and persistent antibody responses^30,31,35^. In addition, they have proven to be highly effective when delivered via mucosal surfaces^22,31,34^. However its mechanism of action is still poorly characterized^2,31,35^.

Herein we show that QB-90, a *Quillaja brasiliensis* leaf-extracted saponin fraction, is efficient in inducing adjuvant effect in mice even when administered up to 24 hours before the inoculation of the antigen, provided the antigen is administered at the same inoculation site. However, when the antigen is inoculated before the adjuvant, such effect is not observed. This interesting finding reveals that QB-90 is capable to promote a local and transient immunocompetent environment. This behavior is also observed in other adjuvants successfully used in human vaccines such as alum^41,42^ and MF59^43^. In conclusion, the findings reported here support that QB-90 is a potent adjuvant with the ability to stimulate a systemic immune response and promote a local and transient immunocompetent environment.

In order to provide a deeper understanding of the mechanisms underlying the generation of the immunocompetent environment, the immune cell recruitment in spleen and dLNs was examined in mice 24 and 48 hpi of QB-90 or IMXQB-90 without antigen. Our results show that either QB-90 or IMXQB-90 inoculation significantly increase spleen cellularity, suggesting a possible rapid drainage from the injection site. Spleen cell influx continues at least up to 48 hours after inoculation of QB-90. In the dLN, both formulations induce an early influx of cells 24 hpi that rapidly decay 24 hours later, being still increased at 48 hpi only in IMXQB-90 inoculated mice. These findings can be explained by the nature of each adjuvant-formulation. As ISCOMs-like structures act as an antigen-delivery system and also are particulate immunostimulatory compounds, they are able to induce a more durable cellular recruitment at the dLNs, than the soluble saponin adjuvant, which is essential to trigger an effective and long-lasting immune response^31^. The above results confirm and extend our previous reports, in which mice treated with ISCOMs-adjuvanted vaccines promoted higher antibodies titers than those inoculated with saponin-adjuvanted vaccines, both in *Q*. *brasiliensis* and commercially available saponins^34^.

When the recruited cells populations were characterized by flow cytometry, an increase in neutrophils, DCs, NKs, B- and T-cells was found in QB-90 and IMXQB-90 treated mice in comparison with the mock control. Interestingly, dLN DCs (CD11b^+^ MHCII^+^) are transiently increased in both inoculated groups at 24 hpi, and their number rapidly decreases for the QB-90 inoculated group, but is still increased in the IMXQB-90 treated group at 48 hpi. On the other hand, splenic DCs behave differently: their number do not increase at 24 hpi, but is significantly augmented in both groups at 48 hpi. The observed behaviour in DCs populations might be explained by the fact that when activated by either QB-90 or IMXQB-90 adjuvants, dLNs DCs may rapidly migrate from dLN to spleen, in order to provide a further activation of immune effector cells. ISCOMs and ISCOMATRIX^TM^ are known to enhance antigen uptake, and induce dLNs DCs activation, resulting in later strong antibody and T cell responses^2,34,37,44^. Our results are in line with previous reports which have proven that ISCOMATRIX^TM^ particles rapidly move to the dLN within the first 2 h after injection in mice, where it can be loaded into dLN-resident DCs and other antigen presenting cells (APCs)^31^. Another work emphasized that DCs and APCs at the injection site also transfer captured ISCOMATRIX^TM^ particles from the injection site into the dLN, thus, providing two waves of vaccine antigen presentation (direct trafficking of adjuvant and via peripheral APC-transport), which result in prolonged antigen presentation^31,35^.

Regarding splenic and dLN neutrophils, QB-90 is able to induce a transient increase 24 hpi, which rapidly decrease and was no longer detectable at 48 hpi. However, IMXQB-90 formulation was able to keep the increase in neutrophils population until, at least, 48 hpi. Although the role of neutrophils in adjuvant activity is not completely clear, this cellular population may play an important role as a vehicle for transporting antigens into the dLN, in order to further influence the activation of different leukocytes, including NK-, B-cells, and DCs^45,46^. At the injection site, neutrophils attract other immune cells by producing increased amounts of chemokines and transport antigens to the dLN^31,47^.

Furthermore, our results show that QB-90 and IMXQB-90 are also able to recruit B and T cells (both CD4^+^ and CD8^+^). Taken together, our results are in good agreement with previous reports that showed that nanoparticulate formulations based in *Q*. *saponaria* saponins increase the number of DCs, NK-, B-, CD4^+^ and CD8^+^ T-cells, and granulocytes in the dLN within 6 h after inoculation, exhibiting a peak 24–48 hpi^4,30,35,44^. Additionally, it has been shown that the cellular influx is transient and reversible, returning to normal levels by 72 hours^35,37^. Regarding NK cells, we show herein that QB-90 and IMXQB-90 promote an increase in this cell population recruitment, in both spleen and dLNs. Interestingly, NK cells are a major source of IFN-γ during early immune responses and are known to enhance antigen-specific T cell responses^48^. In this sense, we detected similar levels of IFN-γ in sera from mice previously sensitized for 24 h with QB-90 or IMXQB-90. However, TNF-α was detected only in mice treated with QB-90, suggesting that IMXQB-90 induces a more controlled inflammatory environment than QB-90.

As mentioned before, our results show a significant increase in DCs recruitment in spleen as well as dLN. In this sense, it has been reported that QuilA^®^ -as well as particulate adjuvants-dramatically enhance IL-1β secretion by DCs^3,5^. Our results show that QB-90-stimulated BMDCs secrete IL-1β in the presence of a TLR agonist such as LPS. Interestingly, BMDCs stimulated solely with IMXQB-90 - without the need of LPS – promote the secretion of IL-1β, and when primed with LPS, IL-1β production is higher than that observed with alum stimulation (a well-known inflammasome activator). In addition, IL-1β secretion observed in IMXQB-90-stimulated DCs is carried out in a caspase-1/11 dependent manner. These results suggest that the canonical-inflammasome pathway is being activated by this adjuvant formulation. When the interaction with innate immunity cellular receptors was assessed *in vitro*, our findings revealed that *Q*. *brasiliensis* saponins, as well as its nanoparticles, interact with macrophages or monocytes in a TLR-independent but in a MyD88-dependent manner. In summary, we suggest that both formulations from *Q*. *brasiliensis* saponins may activate canonical inflammasome pathways, as previously shown for other particulate adjuvants^5,49^ and specifically for ISCOMATRIX^TM^ ^36^. Additionally, the adjuvant effects of QB-90 and IMXQB-90 might depend on the interaction of Caspase-1/11 and MyD88 pathways, consistent with previous reports on saponins from *Q*. *saponaria*^3,4,36^, which have shown to activate innate immunity in a TLR-independent manner and have demonstrated *in vivo* that *Q*. *saponaria* nanoparticles did interact with the adaptor molecule MyD88^44^. Considering these previous reports and our results obtained herein, it can be suggested that MyD88 adaptor molecule is involved in the mechanism of action of *Q*. *brasiliensis* leaf-derived saponins. In fact, it has been reported that ISCOMATRIX^TM^ (formulated with commercially available saponins) induces lysosomal destabilization leading to IL-1β release *in vitro* by APCs through NLRP3 inflammasome^36^. Thus, it has been proposed that ISCOMATRIX^TM^ nanoparticles migrate faster into the cytosol of multiple DCs subsets, promoting the induction of cytokines and chemokines, and bridge innate and adaptive immune responses *in vivo* in a Toll-like receptor-independent but MyD88-dependent manner^44^. To the best of our knowledge, until now, only commercial saponins derived from *Q*. *saponaria* have been described to activate inflammasome pathways^3,4,36,50^. It has been recently reported that soluble QS-21 delivery *in vitro* and *in vivo* induces inflammasome-mediated IL-1β/IL-18 secretion in mice, but the exact mechanism is not clear yet^2,3^. In addition, a novel mechanism of action has recently been proposed for saponins, based on inflammasome activation that enhances antigen cross-presentation by forming intracellular lipid bodies. In this mechanism, lipid bodies escape from endosomes and then translocate antigens for cross-presentation^2^.

Finally, expression profiles of ninety-two immune related genes were assessed by qPCR24 hours after QB-90 and IMXQB-90 sensitization in dLNs. Our results show that some genes encoding for cytokines/chemokines and/or their receptors are differentially regulated by QB-90 or IMXQB-90. QB-90 treatment induces changes in the expression of 41 genes in comparison to control group, while IMXQB-90 alters the expression of 23 genes, being 19 genes in common. These results may suggest that inflammation induced by QB-90 is stronger than that induced by IMXQB-90. For example, QB-90 - unlike IMXQB-90 - induces the up-regulation of genes involved in cellular activation (C*d28*, *Cd3e*, *Cd40*, *and Cd40lg*), co-stimulation (*Cd80*, *Cd86*, *Cd8a*) and transcription factors (*Stat3*, *Stat4*, *Tbx21*). On the other hand, IMXQB-90 do not activate as many pro-inflammatory and activation genes as QB-90, suggesting it may promote less intense and perhaps more controlled inflammatory responses than saponins alone. These findings are in line with results mentioned earlier regarding the cytokine profile elicited by both formulations, in which we found that QB-90 promoted a more proinflammatory environment. In addition, these results correlate with the characteristics of low toxicity and hemolytic activity of these ISCOMs-like formulations^3,27,31,34^. Finally, both formulations yielded a change ≥10-fold in expression of a set of immune-related genes (*Infg*, *Gzmb*, *Ccl2*, *Cxcl10*, *Cxcl11*, *Il5* and *Il6*). Previous reports on early activation of innate cells by ISCOMs or ISCOMATRIX^TM^ showed induction of an array of cytokines and chemokines in serum of vaccinated animals, including pro-inflammatory cytokines (IL-1β, TNF-α and IL-6), as well as chemokines and cytokines involved in macrophage and NK cell activation (CCL3, CCL2, MCP-1, IFN-γ), and/or neutrophil activation and migration (CXCL1, KC, CXCL10, IP-10, G-CSF)^4,37,44^. Interestingly, studies performed with MF59 on human innate immune cells, such as DCs, monocytes, macrophages and granulocytes, showed induction of very similar immune-related genes^51,52^.

Network analysis using the web-based network tool STRING indicates that nearly all differentially expressed genes in the context of QB-90 and IMXQB-90 inoculation are part of well-defined interaction networks. The STRING analysis reveals a strong link with pathways related to immune responses, response to microorganisms and positive regulation of cell proliferation. It is important to highlight that our study was focused only on specific genes, and may have missed possible targets for both formulations. In order to broaden the scope of this assessment, full transcriptome analysis experiments may be conducted to further characterize the transcriptional activity, and enable to create a global picture of the immunocompetent environment created by these promising adjuvants.

In summary, our results show that *Q*. *brasiliensis* saponin-based adjuvants induce an antigen-independent and transient immunocompetent environment. Early cellular responses are observed for both QB-90 and IMXQB-90, at local (dLN) and systemic (spleen) levels. Interestingly, IMXQB-90 elicits a less intense systemic pro-inflammatory response, whilst still being able to activate by itself - at least *in vitro* - the inflammasome through Caspase-1/11 and MyD88 pathways. Moreover, in terms of number of immune related genes, IMXQB-90 also seems to induce a less intense inflammation in the dLN. Nonetheless, when the adaptive immune response was assessed, our previous reports show that both formulations induced similar Th1/Th2 immune profiles, but the nanoparticulate formulation displayed a stronger immune response than the soluble saponin fraction^34^.

Overall, our results reinforce the postulation of using leaf-extracted *Q*. *brasiliensis* saponins and in particular their nanoparticles as potent vaccine adjuvants. We have demonstrated here that they are capable of promoting early immune responses that, considering our previous data, correlate with adequate adaptive immune responses^18,20–23,34^. In addition, *Q*. *brasiliensis* leaves constitute a sustainable and readily renewable alternative source of saponins in relation to *Q*. *saponaria* barks. Last but not least, the results obtained here are particularly relevant, as they contribute to confirm that the mechanism of action of these new adjuvants is similar to that described for commercially available saponins. Thus, these results imply that these novel vaccine formulations may be used to attend uncovered medical needs for animal and human health.

## Materials and Methods

### Ethics statement

Animal manipulation was performed in accordance with “Comisión Honoraria de Experimentación Animal” (CHEA) guidelines and was approved by the Uruguayan University Research Ethics Committee (approval number 070153-000531-13) and Ethics Commission on Animal Experimentation (CEUA) in the “Instituto de PesquisasVeterinárias Desidério Finamor”. Animals were appropriately housed with controlled temperature (22 ± 2 °C) and humidity in a 12/12 h light/dark cycle, with food and water *ad libitum*. All experiments were performed in compliance with the European Convention for the Protection of Vertebrate Animals Used for Experimental and Other Scientific Purposes (European Treaty Series - No. 170 revised 2005) and the procedures of the Brazilian College of Animal Experimentation (COBEA).

### Saponin-derived adjuvants

Leaves from adult plants of *Q*. *brasiliensis* (A. St.-Hil. etTul) Mart. were collected in Canguçu, RS, Brazil (31°23′42″ S–52°40′32″ W) (voucher ICN 142953, deposited at the Herbarium of the Federal University of Rio Grande do Sul). Extraction and purification of saponins were carried out as previously described^17^. IMXQB-90 nanoparticles were prepared by the modified ethanol injection technique^34,53^. Visualization of the nanoparticles was performed by transmission electron microscopy (TEM) (Supplementary Figure).

### Viral antigen preparation and mice immunization

The bovine viral diarrhea virus (BVDV) isolate EVI 001/94 was multiplied in Madin Darby Bovine Kidney cells (MDBK; originally ATCC CCL-22) as described elsewhere^20,24^. The infectious titre of the virus suspension prior to inactivation was 10^7.5^ tissue culture infectious doses per mL. The inactivated virus suspension (referred to as BVDV onwards) was used as antigen for adjuvant testing for all assays.

Conventional female Rockefeller mice (*n* = 5) of the CF-1 breed (5–6 weeks old) were purchased from Fundação Estadual de Produção e Pesquisa em Saúde (FEPPS, Porto Alegre, RS, Brazil). Mice were subcutaneously inoculated (100 µL) in the hind limb twice with a two week interval with either BVDV antigen alone or with adjuvant QB-90 (50 µg). Injections were applied either in the same or opposite member sites, performed simultaneously, or saponin adjuvant was injected 24 or 48 hours prior or post antigen injection. Additionally, at zero hour, the animals were inoculated with formulated (adjuvanted vaccine) or unformulated (two shots; one for antigen and after 5 minutes, one for adjuvant). Mice were bled prior to inoculations (on day 0) and 2 weeks after the second immunization (day 28); sera were kept frozen (at −20 °C) until processed.

### Immunoassays for antibodies and delayed type hypersensitivity (DTH)

Total anti-BVDV IgG1 and IgG2a were determined for each serum samples by ELISA, carried out essentially as previously described^20,24^ using as antigen the BVDV suspension used for mouse immunization. Antibody titres were expressed in OD_492_ nm. Sera were appropriately diluted in PBS-T (1:300 for IgG1 and 1:100 for to IgG2a).

The DTH responses were evaluated in immunized mice on day 28 post first immunization. The BVDV-specific response of each animal was calculated based on values of injected footpad minus the average of the basal swelling^21,24,34^.

### Cell isolation from spleen, draining lymph nodes (dLNs) and flow cytometry analysis

BALB/c mice (8–12 weeks old) were purchased from DILAVE (MGAP-Uruguay) and kept at the Instituto de Higiene (UdelaR). Mice were injected subcutaneously (s.c.) (*n* = 15) at the base of the tail with 25 µL of QB-90 (10 µg) or IMXQB-90 (2.5 µg). The concentration of IMXQB-90 used in this study is defined as the saponin concentration within the particles. Control mice received 25 µL of saline. Spleen and inguinal draining lymph nodes (dLNs) were collected 24 and 48 h post inoculation (p.i.) (*n* = 5). Five 5 animals were used for gene expression studies. From these, dLNs were collected 24 h post adjuvant administration and kept in RNAlater (Ambiom) solution at −80 °C.

Spleen and dLNs were collected in cold PBS and processed by mechanical disaggregation and then filtered through a 100 µm cell strainer (BD Falcon). Cells from the two symmetric dLNsof each mouse were pooled. Splenocytes were incubated in red blood cell lysis buffer, washed with PBS containing 2% FBS and passed through a 100 µm cell strainer. Cells were suspended in FACS buffer (PBS, pH 7.4, 0.5% BSA, 2 mM EDTA and 0.1% sodium azide) and counted with aid of a cell counter (Countess^®^ Automated Cell Counter, Life Technologies^TM^).

For flow cytometry analysis, cells were incubated 20 min at 4 °C with rat serum (10% in FACS buffer) and then transferred to a 96-well microtiter plate and incubated with antibodies for 30 min at 4 °C (5 × 10^5^ cells, 100 µL/well). Antibodies used were anti-mouse CD3:PE (145-2C11), CD4:APC-Cy7 (RM4-5), CD8:PerCP (53-6.7), CD19:FITC (DX5), CD49:FITC (H1.2F3), MHC-II:FITC (I-A/I-E, 2G9), Gr-1:PE-Cy7 (RB6-8C5), CD11c:APC. All staining procedures were conducted on ice and reagents were purchased from Life Technologies. Cell populations were analyzed using a FACS Canto II flow cytometer (BD Biosciences). Retrieved data was analyzed using the FlowJo 7.6.2 software (LLC).

### Cytokine quantification by Cytometric Bead Array (CBA)

Mice were bled 24 and 48 h after sensitized with adjuvant formulation (QB-90 or IMXQB-90) and sera were kept frozen at −80 °C until processed. Cytokines were measured by flow cytometry with aid of the mouse Cytometric Bead Array (CBA) mouse Th1/Th2/Th17 Cytokine Kit (BD Biosciences). Standard curves for each cytokine were plotted and the concentration of each test sample in picograms per milliliter (pg/mL) was calculated using the FCAP software array v1.0.2 (BD Biosciences).

### Murine bone-marrow-derived dendritic cells (BMDC) production and stimulation

C57BL/6 and C57BL/6 Caspase-1/11-deficient mice were purchased from Institut Pasteur de Montevideo. BMDCs were generated by differentiation of bone marrow precursors from 8- to 10-week-old C57BL/6 and KO mice for 10 days in the presence of 20 ng/mL recombinant mouse GM-CSF (PeproTech) as described elsewhere^54^. The cells obtained were between 85 and 95% CD11c^+^. On day 10, cells were plated at 0.4 million per well in 96-well plates and stimuli were added in medium containing 5 ng/mL GM-CSF. BMDC were primed with 10 ng/mL lipopolysaccharide (LPS from *Escherichia coli* O127:B8, Sigma) for 2 hours and then stimulated with different doses (10, 2.5 and 0.5 µg/mL saponins concentration) of QB-90 or IMXQB-90. Alum (Alhydrogel, Sigma) was used as a control for inflammasome activation. After 3 hours, cell response was measured in terms of IL-1β production. Cell culture supernatants were assayed for IL-1β with ELISA kits from Biolegends according to the manufacturer’s instructions. Cell viability was evaluated at the end of the assay by flow cytometry, using Topro-3 staining (Invitrogen).

### Immunostimulation of pattern recognition receptors

To determine if QB-90 or IMXQB-90 were able to activate innate immunity receptors activation, Raw Blue^TM^ (murine macrophages-derived) THP-1 Blue^TM^ (human monocytes-derived) and THP-1 Blue^TM^-defMyD (human monocytes-derived lacking MyD88 gene) reporter cell-lines (InvivoGen) were stimulated with QB-90 or IMXQB-90. These commercial cell-lines used constitutively over express pattern recognition receptors (PRRs), and stably express an NF-κB and AP-1-inducible secrete embryonic alkaline phosphatase (SEAP) reporter gene. Upon TLR stimulation this cells activate transcription factors and subsequently the secretion of SEAP, which is detectable by the addition of QUANTI-Blue^TM^.

Raw Blue™ (1 × 10^5^ cells/mL), THP1-XBlue™ and THP1-XBlue™-defMyD (1 × 10^6^ cells/mL) were plated in 96-well cell-culture plates (Greiner) following manufacture’s recommendations (InvivoGen^®^). Cells were stimulated with different concentrations of QB-90 or IMXQB-90 (6.250, 1.562, 0.390, 0.097, 0.0244, 0.006 µg/mL of saponins), and cultured overnight at 37 °C and 5% CO_2_. Each sample was tested in triplicate. As positive control L-Ala-γ -D-Glu-mDAP (Tri-DAP 10 µg/mL, InvivoGen^®^) was used for THP-1 cells, and LPS (1 µg/mL, Sigma) for Raw Blue^TM^ cells. In all cell lines Ca^2+^ and Mg^2+^ free phosphate-saline buffer (PBS) was used as a negative control, and the ethanolic stock solution used for the IMXQB-90 formulation was included as vehicle control. After incubation, supernatants were measured using QUANTI-Blue^TM^ in accordance with manufacturer’s specifications. Plates containing supernatants and QUANTI-Blue^TM^ were incubated during 3 hours at 37 °C and SEAP activity, representing activation of NF-κB/AP-1, was measured on a Tecan Sunrise^TM^ microplate reader (TECAN) using Magellan Data Acquisition & Analysis Soſtware version 7.2^©^.

### Cell cytotoxicity assay

Cytotoxicity was determined using the MTT assay. Briefly, reporter cell lines RawXBlue^TM^ and THP1-XBlue™ (InvivoGen^®^) were seeded at a concentration of 4.0 × 10^4^ per well on 96-well cell-culture plates (Greiner) and maintained at 37 °C under a humid atmosphere with 5% CO_2_. After 18 h, the medium was removed and 100 µL of culture medium containing different concentrations of either QB-90 or IMXQB-90 (6.250, 1.562, 0.390, 0.097, 0.0244, 0.006 µg/mL of saponins) were added to each well. Six replicates of each sample were performed. Plates were incubated overnight at 37 °C and 5% CO_2_. After incubation, 50 µL of 5 mg/mL of MTT (Sigma) were added to each well and the cells were incubated 4 hours. The plates were centrifuged (1500 × g for 5 min) and the supernatants containing untransformed MTT were carefully removed. 50 µL of 20% sodium dodecyl sulfate (SDS, Sigma) were added to each well in order to solubilize the formazan crystals, and the OD_540_ nm was measured in an ELISA reader (Tecan Sunrise^TM^). Results were expressed as the percentage of viability, considering OD of untreated control cells as 100% of viability.

### RNA isolation and immune-related gene expression

Changes in mouse immune-related gene expression in dLNs from mice inoculated with either QB-90 or IMXQB-90 preparations were detected using a TaqMan^®^ Mouse Immune Array by qPCR with the 7500 Real-Time PCR System (Applied Biosystems). For these studies, RNA was isolated from dLNs at 24 h post adjuvant administration (or mock inoculated with saline solution). Each experimental sample represents two pooled dLN collected from mice (n = 5). Prior to RNA isolation, dLNs were homogenized in 1 mL of TRIzol (Ambiom). RNA was further purified using Pure Link RNA mini Kit (Ambiom). RNA integrity was checked on Bioanalyzer (Agilent) and quantified by fluorometry (Qubit, Invitrogen). cDNA was synthetized by High-Capacity cDNA Reverse Transcription Kit (Thermo Fisher Scientific). Fold change was calculated using the Pfaffl method (2^−ΔΔCt^) for dLN from QB-90 and IMXQB-90 and being compared with the mock group^55^. The online software STRING (combined score >0.4) was carried on to perform protein interaction network analysis^56^.

### Statistical analysis

The significance of differences was assessed using nonparametric Kruskal-Wallis test with Dunn’s post test. Students T- test was used for evaluation BMDC experiment. GraphPad Prism version7.00 (GraphPad Software, Inc.) was used for data analysis. Results are expressed as the mean or median value from individuals in each group ± SEM or box-and-whiskers. A *p* value equal or less than 0.05 was considered statistically significant.

## Electronic supplementary material

Supplementary Figure 1. Transmission electron microscopy (TEM) of IMXQB-90.
