# Supplementary material for: Quillaja brasiliensis saponin-based nanoparticulate adjuvants are capable of triggering early immune responses
Source: Sci Rep. 2018 Sep 11;8:13582. doi: 10.1038/s41598-018-31995-1 (PMC6134118; doi:10.1038/s41598-018-31995-1)
Supplement: Supplementary file 1 — Supplementary Figure 1. Transmission electron microscopy (TEM) of IMXQB-90. [file 41598_2018_31995_MOESM1_ESM.docx]

***Quillaja brasiliensis* saponin-based nanoparticulate adjuvants are capable of triggering early immune responses.**

Samuel Paulo Cibulski^1^, Mariana Rivera-Patron^2^, Gustavo Mourglia-Ettlin^3^, Cecilia Casaravilla^3^, Anna Carolina Alves Yendo^4^, Arthur Germano Fett-Neto^4^, José Alejandro Chabalgoity^2^, Maria Moreno^2^, Paulo Michel Roehe^1^ and Fernando Silveira^2,^*

^1^Departamento de Microbiologia, Laboratório de Virologia, Universidade Federal do Rio Grande do Sul (UFRGS), Porto Alegre, RS, Brazil.

^2^Departamento de Desarrollo Biotecnológico. Instituto de Higiene – Facultad de Medicina, Universidad de la República (UdelaR). Av. Alfredo Navarro 3051. CP. 11600, Montevideo, Uruguay.

^3^Área Inmunología, Departamento de Biociencias/Instituto de Química Biológica – Facultad de Química/Ciencias, Universidad de la República (UdelaR). Av. Alfredo Navarro 3051. CP. 11600, Montevideo, Uruguay.

^4^Laboratório de Fisiologia Vegetal, Centro de Biotecnologia e Departamento de Botânica, Universidade Federal do Rio Grande do Sul (UFRGS), Porto Alegre, RS, Brazil.

*Corresponding author. E-mail address: [fernandosilveiragonzalez@gmail.com](mailto:fsilveira@higiene.edu.uy)

**Supplementary Figure 1. Transmission electron microscopy (TEM) of IMXQB-90.** Microphotography of IMXQB-90 prepared with a purified saponin-fraction from *Quillaja brasiliensis* (QB-90) by the ethanol injection technique. QB-90 formulation with 3:2:5 relative proportions of saponins:cholesterol:phosphatidylcholine rendered mostly ISCOMATRIX particles with an average diameter of 47 nm (overall range of 40–50 nm).
